# A feature recognition and detection algorithm for pine wilt disease trees based on FLMP-YOLOv8

**DOI:** 10.1371/journal.pone.0337109

**Published:** 2025-12-04

**Authors:** Xiaozhou Feng, Xiaoting Zhao, Hua Shi, Chaobo Chen, Yufen Xie, Mengyan Liu, Hao Hu, Hui Guo, Pengxiang Xue

**Affiliations:** 1 College of Electronic and Information Engineering, Xi’an Technological University, Xi’an, China; 2 College of Sciences, Xi’an Technological University, Xi’an, China; 3 Institute of Forest Protection, Shaanxi Academy of Forestry Sciences, Xi’an, China; University of Agriculture Faisalabad, PAKISTAN

## Abstract

Pine wilt disease, a highly contagious forest disease caused by the pine wood nematode and primarily transmitted via its insect vector, the pine sawyer beetle (Monochamus spp.), poses a significant threat to forest ecosystems. Accurate detection of infected trees is vital for effective prevention and control. This study pioneers the detection of pine wilt disease-infected trees in the China’s Qinba Mountain region, where the complex terrain and uneven forest distribution thinder feature extraction of diseased trees. To address data collection challenge, this paper proposes a novel feature recognition and detection method for pine wilt disease-infected trees based on an FLMP-YOLOv8 algorithm. The enhanced features include: first, integrating FasterBlock module into the backbone and neck networks of YOLOv8 to, boost the model’s feature extraction capability and reduce complexity, thereby achieving a balance between detection efficiency and accuracy. Second, a Large Separable Kernel Attention (LSKA) mechanism is incorporated into the Spatial Pyramid Pooling-Fast (SPPF) module of YOLOv8, improving the model’s ability to perceive fine details of diseased trees and reducing interference from other elements in the forest. Finally, the MPDIoU loss function is adopted for bounding box regression, enhancing the precision of localization. Experimental results on a self-constructed dataset demonstrate the improved model efficacy, achieving 92.0% precision, 80.8% recall, 87.0% mean Average Precision (mAP@0.5), and 81.79 FPS detection speed. Compared to the original YOLOv8 model, the improved algorithm shows increases of 2.2% in precision, 0.6% in recall, and 2.0% in mAP@0.5, with a detection speed improvement of 65.48 FPS. This study provides a more reliable and cost-effective method for detecting trees infected with pine wilt disease.

## Introduction

Forest pest and disease control pose significant challenges, with pine wilt disease (PWD) [1–6], caused by Bursaphelenchus xylophilus, being among the most devastating. Known as the “cancer of pine trees,” it spreads rapidly, exhibits high pathogenicity, and remains extremely difficult to eradicate. Originating in North America, PWD has now spread to countries including Japan, South Korea, China, Portugal, and Spain, becoming a global forestry disaster. Given its rapid transmission and severe ecological impact, developing efficient and accurate detection technologies for infected trees is of great scientific and practical significance [7,8].

Traditional detection of PWD-infected trees relied on field surveys by forestry personnel. However, manual inspections are costly, labor-intensive, and highly subjective. With recent advances in remote sensing and computer vision, intelligent detection methods based on machine learning and deep learning have emerged, offering new technical pathways for large-scale and precise forest disease monitoring.

Early machine learning approaches typically used manually extracted features—such as color, texture, and morphology—combined with classifiers like Support Vector Machines (SVM) or Random Forests (RF). Studies in Portugal, Japan, and China reported overall accuracies above 0.9 using multispectral or hyperspectral data. These methods are interpretable and effective with small samples but depend heavily on costly sensors and manual feature engineering, limiting their generalization in diverse forest environments [9–12].

The rise of deep learning has transformed forest disease detection, allowing end-to-end learning and automatic extraction of hierarchical features from large datasets. Deep learning–based object detection algorithms are generally divided into two-stage and one-stage frameworks. Two-stage detectors (e.g., Faster R-CNN, Cascade R-CNN) achieve high precision through region proposal and classification stages, suitable for small or sparse targets. One-stage detectors (e.g., YOLO, SSD) directly predict bounding boxes and classes in a single step, achieving faster inference for large-scale aerial imagery.

Recent studies have improved both frameworks by incorporating attention mechanisms, transformer architectures, or custom loss functions. Examples include enhanced Mask R-CNN models using ConvNeXt or PA-FPN modules for high-resolution imagery, lightweight YOLO variants using attention mechanisms such as CBAM and Coordinate Attention, and Vision Transformer–based detectors like Light-ViTeYOLO for small or scale-varying targets. These innovations balance accuracy, computational efficiency, and real-time applicability, advancing PWD detection from manual to fully automated levels [13–17].

Despite significant progress, current PWD detection research remains concentrated in East Asia and parts of Europe, with limited studies in the complex mountainous regions of western China. The Qinba Mountains, characterized by rugged terrain, large elevation gradients, and frequent lighting variations, present severe challenges for UAV image acquisition and analysis [18,19]. Additionally, the spectral similarity between diseased pines and surrounding bare soil leads to frequent misdetections using conventional RGB-based models [20].

To address these challenges, this study proposes an improved YOLOv8-based model, FLMP-YOLOv8, for detecting PWD-infected trees in the Qinba Mountains using RGB UAV imagery [21–23]. The model introduces several architectural and functional enhancements:

(1) The FasterBlock module replaces the Bottleneck module in both the backbone and neck networks, optimizing feature extraction and reducing computational complexity while maintaining accuracy.

(2) The LSKA attention mechanism is integrated into the SPPF module to improve multi-scale feature perception, effectively distinguishing diseased trees from complex forest backgrounds.

(3) The MPDIoU loss function replaces the standard CIoU to improve bounding box regression accuracy and convergence speed, enhancing detection robustness under varying illumination and texture conditions.

This approach retains the economic advantage and simplicity of RGB imagery while achieving high detection accuracy in complex natural environments. Overall, the proposed method provides a cost-effective, fast, and robust solution for UAV-based PWD detection in mountainous forest regions, contributing to intelligent forestry management and early warning systems.

## Materials and methods

### YOLOv8 baseline model

In UAV-based object detection of pine wilt disease-infected trees across the Qinling Mountains [24–26], conventional object detectors often face difficulties due to the limited pixel representation and weakly discriminative features of diseased tree regions. This challenge challenge is particularly pronounced in the ecologically complex Qinba Mountain forests, where infected trees demonstrate near-indistinguishable colorimetric, geometric, and textural similarities to surrounding terrain features (including soil strata, rock outcrops, and leaf litter). The Qinling range’s pronounce topographic relief further complicate detection with non-uniform illumination in aerial imagery. The synergistic interplay of these factors elevates the complexity of feature extraction and detection of pine wilt disease symptomatic trees to a particularly challenging level.

**Fig 1:**
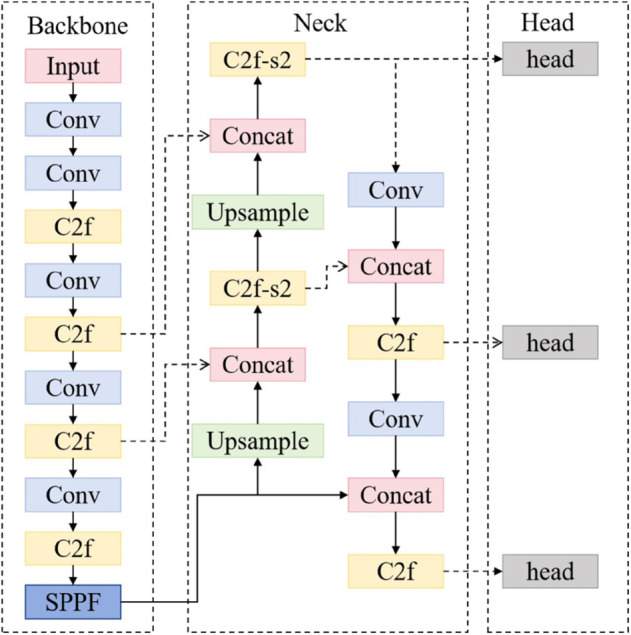
YOLOv8 model architecture diagram.

YOLO, an object detection algorithm originally proposed by Redmon *et al* [27–29]., performs detection tasks within a unified network architecture. Through iterative optimizations, YOLOv8 has achieved remarkable advances in both speed and accuracy and is currently implemented within the PyTorch framework. Building on enhanced YOLOv8 models [30–32], this study develops an improved YOLOv8-based detection algorithm specifically tailored for identifying and characterizing infected trees in the complex terrain of the Qinling Mountains. The proposed algorithmic enhancements aim to significantly improve the monitoring and prevention efficiency of forest pest and disease management. The YOLOv8 model structure is shown in Fig 1

As shown in the Fig 1, YOLOv8 adopts a single-stage detection architecture, integrating a lightweight backbone network with an efficient detection head to achieve fast object detection and accurate localization. It extracts image features through the feature extraction network, enhances the feature representation ability via the feature enhancement network, and makes target predictions in the detection head. This structure enables YOLOv8 to detect objects in images efficiently and accurately.

### FLMP-YOLOv8 model

**Fig 2:**
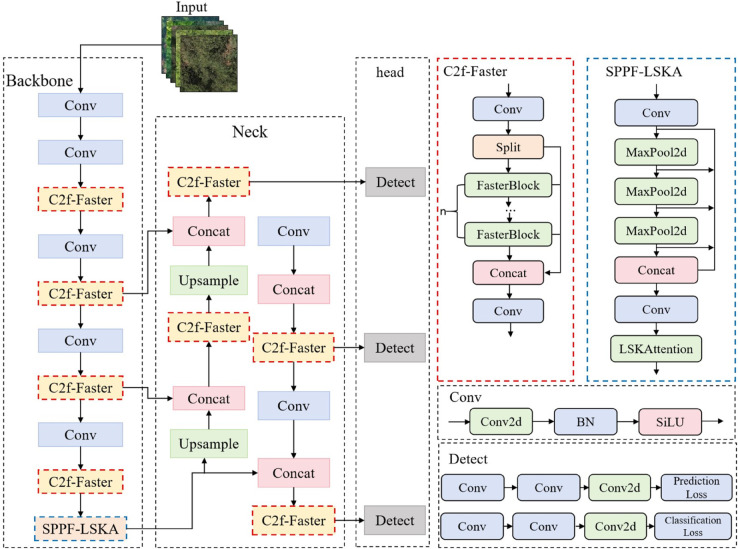
FLMP-YOLOv8 architecture diagram.

To meet the practical requirements of pine wilt disease tree detection, the YOLOv8 model has been improved [33,34]. The Bottleneck in the C2f (CSP Bottleneck with 2 Convolutions) module is replaced with the FasterBlock structure to enhance the model’s detection speed. The LSKAttention mechanism is integrated into the Spatial Pyramid Pooling-Fast (SPPF) layer to enhance multi-scale feature aggregation. Additionally, the MPDIoU loss function is employed for bounding box regression, ensuring that the predicted boxes for diseased tree targets more closely align with the ground truth, thereby improving the overall detection accuracy of the model. The architecture of the proposed FLMP-YOLOv8 object detection model is illustrated in Fig 2.

As shown in Fig 2, the red dashed box indicates the position and specific structure of the C2f-Faster module in the improved model, while the blue dashed box indicates the position and specific structure of the SPPF-LSKA module in the improved model.

#### FarsterBlock module.

**Fig 3:**
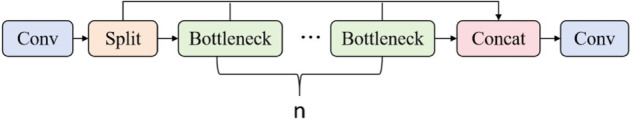
C2f structure.

**Fig 4:**
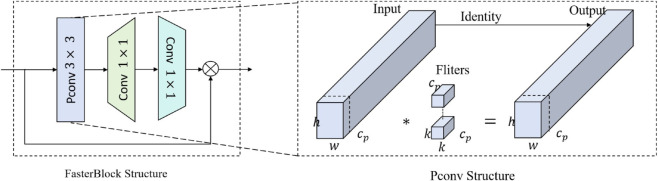
FarsterBlock structure.

The integration of multi-level Bottleneck structure with the C2f module of YOLOv8 has a relatively large number of parameters and high computational complexity [35]. To enhance the model’s performance, this study replaces the Bottleneck structure in the C2f module of both the backbone and neck networks with a more lightweight FarsterBlock structure [36–38]. The structure diagrams of C2f and FarsterBlock are shown in Figs 3 and 4, respectively.

As shown in Fig 3, the C2f module has more parameters and a larger model size, resulting in slower detection speed for pine wilt disease tree targets. The Bottleneck, as the main structure of C2f, primarily uses a pointwise convolution (PW-Conv) and a 3×3 convolution. The computational complexity of the convolutions in the Bottleneck module is as shown in (1).

$$F_{\text{Bottleneck}}=2\times h\times w\times c^{2}\times k^{2}\;=2\times 3\times 3\times h\times w\times c^{2}\;=18\times h\times w\times c^{2}$$ (1)

Specifically, h denotes the height of the feature map, w represents the width, c refers to the number of input or output channels, and k indicates the kernel size, which is 3×3 in this case.

The FasterBlock, serving as the core component of FasterNet [39,40], is illustrated in Fig 4. It consists of one partial convolution (PConv) and two pointwise convolutions (PWConvs). The PConv operation is selectively applied to one-quarter of the input feature channels, while the remaining three-quarters are retained unchanged to facilitate subsequent feature fusion. The computational cost of the PConv operation can be expressed as $h\times w\times k^{2}\times c_{p}^{2}$ $\left(c_{p}=\frac{1}{4}c\right)$. Following this, the FasterBlock utilizes PWConv to fully extract channel features. The overall computational cost of FasterBlock is as shown in (2).

$$F_{\text{FasterBlock}}=h\times w\times c_{p}^{2}\times k^{2}+2\times h\times w\times c^{2}\times k_{1}^{2}\;=h\times w\times \left(\frac{1}{4}c\right)\times \left(\frac{1}{4}c\right)\times 3\times 3+2\times h\times w\times c^{2}\times 1\times 1\;=\frac{9}{16}h\times w\times c^{2}+2\times h\times w\times c^{2}\;=\frac{41}{16}h\times w\times c^{2}$$ (2)

*k*_1_ denotes a convolutional kernel with a size of 1×1.

By comparison, the total computational cost of the FarsterBlock is lower than that of the Bottleneck. To achieve a more lightweight model with higher detection accuracy, this study introduces the FarsterBlock to replace the Bottleneck module. The improved C2f module is accordingly named the C2f-Faster module.

#### Large separable kernel attention.

**Fig 5:**
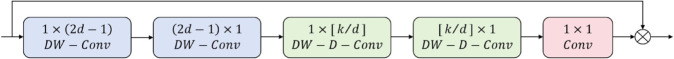
LSKA module structure diagram.

To enhance the performance of the detection model for UAV aerial images with diverse forest backgrounds, the LSKA attention mechanism is integrated into the SPPF module of YOLOv8 [41–43]. This mechanism enables the model focus more on important information in local regions during feature extraction, improving its ability to perceive details, especially when dealing with targets that are similar to the background. The mechanism assigns different weights to features at different spatial locations, enabling the model to recognize and accurately locate targets such as diseased trees in complex backgrounds, thereby reducing the impact of background interference. This improves the detection accuracy and robustness for pine wilt disease-infected trees. The structure of the LSKA attention mechanism is shown in Fig 5.

As shown in Fig 5, the LSKA module enhances the model’s receptive field by decomposing the conventional 2D convolution kernel into two sequential 1D convolution kernels. This enables the model to capture multi-scale information, from local to global features. Consequently, it not only helps the model adapt to targets of varying sizes but also improves its ability to capture target details in complex forest backgrounds, while effectively reduces parameters and computational complexity. Additionally, LSKA dynamically adjusts parameters d (the receptive field expansion factor) and k (the kernel size), enabling flexible control over the receptive field and enhancing the model’s adaptability to diverse scenes and targets. In summary, the LSKA module integrates standard depthwise convolution (DW-Conv), dilated depthwise convolution (DW-D-Conv), and 1×1 channel convolution to improve detection of pinewood nematode disease trees in varied forest backgrounds.

The output of the LSKA module is as shown in (3)–(6).

$${\bar{Z}}^{c}=\sum_{H,W}W_{\left(2d-1)\times (2d-1\right)}^{c}*F^{c}$$ (3)

$$Z^{c}=\sum_{H,W}W_{\left(k/d)\times (k/d\right)}^{c}*{\bar{Z}}^{c}$$ (4)

$$A^{c}=W_{1\times 1}*Z^{c}$$ (5)

$${\bar{F}}^{c}=A^{c}⊗F^{c}$$ (6)

Where, * represents convolution, ⊗ represents the Hadamard operation, *d* represents the dilation rate, ${\bar{Z}}^{c}$ represents the output of the depthwise convolution with a kernel size of (2d−1)×(2d−1), *Z*^*c*^ represents the output of the depthwise dilated convolution with a kernel size of (k/d)×(k/d), and the attention map *A*^*c*^ is obtained through a 1×1 convolution. Finally, the output of the LSKA module, ${\bar{F}}^{c}$, is the result of the Hadamard operation between the attention map *A*^*c*^ and the input feature map *F*^*c*^.

#### Optimizing the loss function.

In aerial forest images, diseased tree targets often exhibit significant varaiations in the shape and orientation of the bounding boxes due to differences in camera angle, lighting conditions, and shooting position. Common regression loss functions include DioU, CIoU, and GioU. However, when the aspect ratios of the predicted box and the ground truth bounding box are identical but their width and height values are entirely different, most existing bounding box regression loss functions continue to detect correctly. To address this issue, this paper adopts the MPDIoU regression loss function [44,45].The function aims to accurately measure the similarity between the predicted box and the ground truth bounding box, optimizing localization accuracy of the bounding boxes and providing reliable support for object detection and instance segmentation tasks. The formulation of the MPDIoU loss function is defined in equations 7–(9).

$$M_{\text{plotU}}=\frac{A_{\text{UB}}}{w\cdot A_{\text{UB}}+h\cdot l}-c_{1}\cdot \frac{d_{1}^{2}}{w^{2}+h^{2}}-c_{1}\cdot \frac{d_{2}^{2}}{w^{2}+h^{2}}$$ (7)

$$d_{1}^{2}=(x_{1}^{\text{b}}-x_{1}^{\text{t}}{)}^{2}+(y_{1}^{\text{b}}-y_{1}^{\text{t}}{)}^{2}$$ (8)

$$d_{2}^{2}=(x_{2}^{\text{b}}-x_{2}^{\text{a}}{)}^{2}+(y_{2}^{\text{b}}-y_{2}^{\text{a}}{)}^{2}$$ (9)

Here, the superscripts *a* and *b* represent the annotated box and the predicted box, respectively; *A* is the ground truth box; *B* is the predicted box; the subscripts 1 and 2 denote the top-right and bottom-left corners of the corresponding boxes, respectively. $\frac{d_{1}^{2}}{w^{2}+h^{2}}$ and $\frac{d_{2}^{2}}{w^{2}+h^{2}}$ represent the normalization of the diagonal distance to facilitate a comparison of the diagonal distance with the IoU on the same scale. *c*_1_ is used to adjust the weight of the diagonal distance in order to better measure the impact of positional offset on the IoU.

### Experiment environment and dataset

#### Experiment environment and parameter settings.

The experiment was conducted using the PyTorch deep learning framework on a Windows 10 system, with specific configuration outlined in Table 1.

**Table 1:** Experimental environment configuration.

Component	Specification
Operating System	Windows10
CPU	12th Gen Intel(R) Core(TM) i7-12700
RAM	32.0GB
GPU	NVIDIA GeForce RTX 4060 Ti
Pytorch	3.5.1
Torchvision	0.20.1
CUDA	12.7
Python	3.12.7

To ensure the comparability of the experimental results, all experiments in this paper used a unified training configuration, with the specific training parameters shown in Table 2.

**Table 2:** Experimental parameter configuration.

Parameters	Setup
Epoch	150
Batch Size	4
ImgSize	640×640
Initial Learning Rate	0.01
Final Learning Rate	0.001
Patience	10

#### Pinewood nematode disease tree dataset.

The study utilized data collected from pine forests in the Qinba Mountain area between June and November 2024, primarily through drone aerial photography. To ensure data representativeness and reliability, a rigorous aerial imaging plan was established. The drone flight altitude was set at multiple levels ranging from 50 meters to 120 meters, adjusted according to the forest density and terrain features.

**Fig 6:**
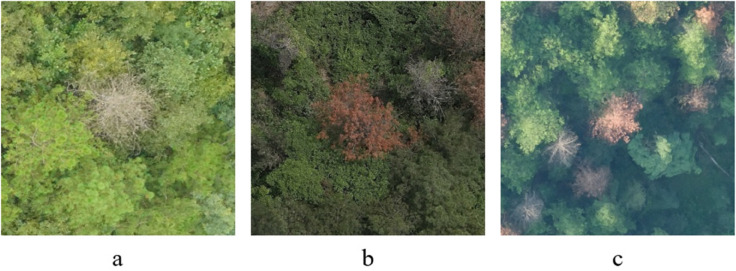
Example sample image of pine wood nematode-infected trees.

The obtained high-resolution image data has been segmented and filtered, resulting in a total of 1,200 images with a resolution of 640×640 pixels. In the dataset, pinewood nematode disease trees are specifically characterized by features such as tree wilting and discoloration. As shown in Fig 6, examples of pinewood nematode disease trees under different segments and lighting conditions are presented.

Fig 6 illustrates the symptoms of pine wilt disease under different lighting conditions. (a) shows trees in the late stage of infection, where the crowns are completely wilted and appear whitish. (b) contains both mid- and late-stage infected trees, with the former displaying reddish crowns and the latter exhibiting white, fully wilted crowns. (c) presents trees at various stages of infection, where early-stage trees appear light green, mid-stage trees turn red, and late-stage trees become white, clearly demonstrating the progressive characteristics of the disease.

**Fig 7:**
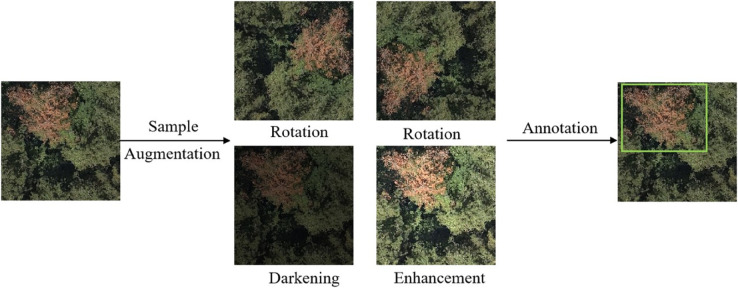
Sample augmentation and annotation illustration.

To improve the model’s generalization ability and enhance its recognition of diseased trees from different angles, data augmentation operations such as multi-angle rotation and brightness adjustment were applied to the original dataset. This significantly increased the number and diversity of the samples. The illustration of the sample augmentation operations is shown in Fig 7.

Employing the online annotation tool Makesense in conjunction with forestry experts, the targets were selected using rectangular boxes, and the corresponding category information was labeled for each selected diseased tree. The label for the diseased tree area was set as “sicktree.” The annotation results were saved in .txt format. To enhance the model’s generaliz ability, data augmentation operations such as multi-angle rotation and brightness adjustment were applied to the original dataset, significantly increasing the number and diversity of training samples. A total of 5,436 pinewood nematode disease tree image samples were obtained. These samples were split into training set and test set in a 7:3 ratio. The annotation illustration is shown in Fig 7.

### Evaluation metrics

The experimental results select Precision (P), Recall (R), Mean Average Precision (mAP), and Frames Per Second (FPS) as evaluation metrics. The formulas for calculating Precision (P) and Recall (R) are show in (10) and (11).

$$P=\frac{TP}{TP+FP}$$ (10)

$$R=\frac{TP}{TP+FN}$$ (11)

In the formulas, TP (true positive) represents the number of targets correctly predicted by the model; FP (false positive) refers to the number of targets incorrectly predicted by the model; FN (false negative) represents the actual diseased tree targets that the model failed to predict.

The formula for calculating Average Precision (AP) is as show in (12).

$$AP=\frac{1}{m}\sum_{n=1}^{m}(P(r)\Delta R(r))=\int_{0}^{1}P(r)\text{d}R$$ (12)

Here, *m* represents the number of positive samples, *P*(*r*) is the proportion of positive samples in the top *r* retrieved results, ΔR(r) is the change in recall rate relative to *r* in the top *r* retrieved results, and *P*(*R*) represents the precision (*P*) at recall rate (*R*). *AP* represents the average value under the precision-recall (*P*-*R*) curve, indicating precision at different recall rates. To calculate *AP*, a numerical integration method is used to sum the *AP* for all categories, and the average is taken to obtain the mean Average Precision (*mAP*), which represents the model’s average precision. The formula for *mAP* is as shown in (13).

$$mAP=\frac{1}{n}\sum_{C=1}^{n}AP$$ (13)

Here, *AP*_*i*_ is the average precision for the *i*-th category.

*mAP*_50_ represents the *mAP* value at a 50% IoU threshold. The formula for *mAP*_50_ is as shown in (14).

$$mAP_{50}=\frac{1}{n}\sum_{i=1}^{n}AP_{i}$$ (14)

FPS (Frames Per Second) represents the number of image frames processed per unit of time. The higher the value, the faster the model’s detection speed.

## Results

### Comparative experiments

To evaluate the effectiveness of the proposed improvements in this study, comparative experiments were designed based on various object detection models, diverse attention mechanism modules, and different C2f variant modules.

The proposed algorithm was compared with other algorithms in the YOLO series. YOLO algorithms, classic models in the field of object detection, offer high detection accuracy and real-time performance, making them widely used in various computer vision tasks. In this experiment, three representative algorithms from the YOLO series—YOLOv3, YOLOv5, and YOLOv8—were selected as comparison models. The experimental results are shown in Table 3.

**Table 3:** Comparative results of different detection algorithms.

models	P(%)	R(%)	$mAP_{50}$(%)
YOLOv3	78.3	65.7	68.7
YOLOv5	86.1	79.0	84.6
YOLOv8	89.8	80.2	85.0

As shown in Table 3, the YOLOv8 baseline model used in this study outperforms other classic YOLO series models in various detection performance metrics for pinewood nematode-infected trees under diverse forest backgrounds.

Under the same experimental conditions, different attention mechanisms—including ECA, GAM, PSA, and LSKA—were introduced based on the YOLOv8 model to compare their impact on detection performance. The comparative results of different attention mechanisms are shown in Table 4.

**Table 4:** Comparative results of different attention mechanisms.

Models	P(%)	R(%)	$mAP_{50}$(%)
YOLOv8	89.8	80.2	85.0
YOLOv8+ECA	88.5	80.6	85.7
YOLOv8+PSA	90.4	78.3	84.8
YOLOv8+GAM	90.6	80.3	86.1
YOLOv8+LSKA	88.3	81.6	86.2

As shown in Table 4, compared with other models, YOLOv8+LSKA exhibits the best performance in both recall and mean average precision, indicating that the YOLOv8+LSKA model has greater advantages in reducing missed detections and improving overall detection performance for pinewood nematode-infected trees.

Under the same experimental conditions, comparative experiments were conducted by replacing the C2f module in the YOLOv8 model with C2f-Saconv, C2f-Ghost, C2f-MLLABloc, and C2f-Faster. The results of different C2f variant modules are shown in Table 5.

**Table 5:** Comparative results of different C2f variant modules.

Models	P(%)	R(%)	$mAP_{50}$(%)	parameters
YOLOv8	89.8	80.2	85.0	3101043
YOLOv8+C2f-GhostConv	88.3	82.6	86.3	2368077
YOLOv8+C2f-MallerBlock	90.9	80.4	85.7	3150113
YOLOv8+C2f-Faster	84.7	76.0	81.8	2387043
YOLOv8+C2f-LSKA	91.6	81.1	86.3	2895434

As shown in Table 5, compared with other models, the YOLOv8+C2f-Faster model improves the mean average precision and maintains computational efficiency, achieving a good balance between the two. Therefore, replacing C2f with C2f-Faster offers the best overall performance.

### Ablation study

To evaluate the effectiveness of each improvement, YOLOv8 was adopted as the baseline model, and six ablation experiment groups were designed under identical experimental conditions. Experiment Group 1 is the original YOLOv8 baseline model; Experiment Group 2 introduces the C2F-faster module to verify its efficiency improvement in feature extraction; Experiment Group 3 introduces the SPPF-LSKA structure to evaluate its enhancement effect on multi-scale feature fusion; Experiment Group 4 introduces the MPDIou loss function; Experiment Group 5 combines C2F-faster and SPPF-LSKA to analyze their synergistic effect; and Experiment Group 6 comprehensively verifies the combined optimization effect of the three improvement strategies. The experimental results provide performance data for different algorithms.

**Fig 8:**
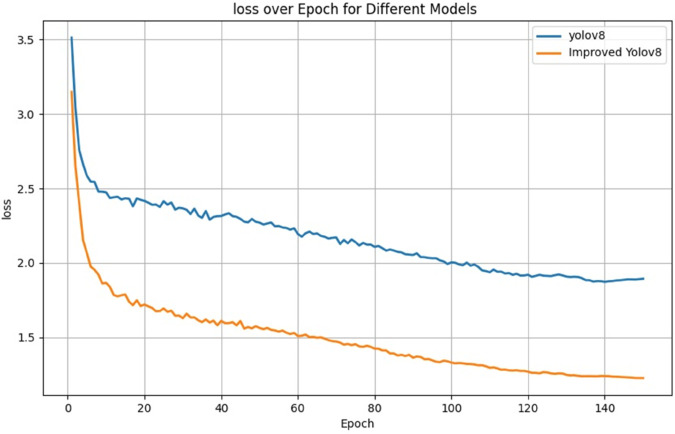
Comparison of loss function curves before and after improvement.

**Fig 9:**
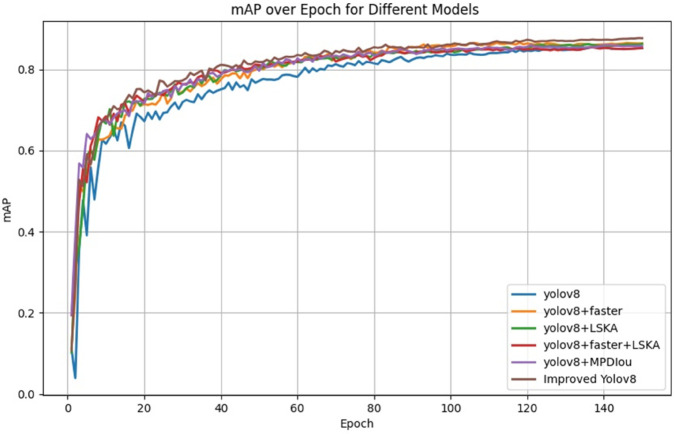
Comparison of mAP curves in ablation experiments.

The FLMP-YOLOv8 model constructed by stacking multiple improved modules on the baseline YOLOv8 architecture to verify their synergy and complementarity. The results are quantitatively discussed in terms of the changes in evaluation metrics across the six models. The experimental results are shown in Table 6, with the loss function curves before and after the improvements shown in Fig 8, and the mean average precision (mAP) curves for the six models presented in Fig 9.

**Table 6:** Ablation experiment results table.

Experiment Group	Models	P(%)	R(%)	$mAP_{50}$(%)	parameters	FPS(/s)
1	Yolov8	89.8	80.2	85.0	3011043	16.31
2	Yolov8+C2fFaster	91.7	81.1	86.3	2305843	34.6
3	Yolov8+LSKA	88.3	81.6	86.2	3084003	20.97
4	Yolov8+MPDIoU	89.6	81.3	85.9	3011043	21.61
5	Yolov8+C2fFaster+LSKA	88.7	80.6	85.4	2378803	81.52
6	FLMP-YOLOv8	92.0	80.8	87.0	2378803	81.79

As shown in Table 6, Experiment Group 1 is the YOLOv8 baseline model, without any additional modules. The precision is 87.7%, recall is 80.2%, and the average precision is 84.7%.

Experiment Group 2 replaces the Bottleneck in the C2f module of the YOLOv8 backbone and neck network with the FasterBlock structure. This reduces the number of parameters while enabling more efficient multi-scale feature fusion and processing, effectively detecting targets of different sizes. Compared to the baseline model, its precision increases by 1.9%, recall increases by 0.9%, and average precision improves by 1.6%.

Experiment Group 3 integrates the LSKA attention mechanism into the SPPF module of YOLOv8 to capture multi-scale features through pooling at different scales. Compared to the baseline model, performance indicators improve, but compared to Experiment 1, network complexity increases, the number of parameters grows, and detection speed decreases. Experiment Group 4 adopts the MPDIoU loss function, which improves the detection accuracy of the diseased trees by incorporating the distance and intersection between polygons, leading to overall performance improvement.

Experiment Groups 5 and 6 are combinations of the improvement modules. The experimental results confirm that each improvement module is effective for the model. The ablation experiment evaluates all improvement strategies in sequence, and the results show that the final improvement method enhances all indicators, achieving a precision of 92.0%, recall of 80.8%, and average precision of 87.0%. Compared to the baseline model, precision increases by 2.2%, recall improves by 0.6%, and average precision increases by 2.0%. Meanwhile, the model’s parameter count is reduced, improving detection efficiency.

Since the Improved YOLOv8 model introduces the MPDIoU loss function, as shown in Fig 8, its overall loss performance is better than the YOLOv8 baseline model. Additionally, the Improved YOLOv8 exhibits a faster loss decrease and a smoother loss curve, indicating that the Improved YOLOv8 model has a faster convergence speed and higher stability.

As shown in Fig 9, the average precision (mAP) values of different models during the training process are plotted as epochs increase. The results indicate that the Improved YOLOv8 maintains a high mAP throughout the entire training process and stabilizes during later epochs, outperforming the original YOLOv8 model and other ablation experiment models. Therefore, the Improved YOLOv8 demonstrates superior detection performance in the pine wood nematode disease tree detection task in this study.

**Fig 10:**
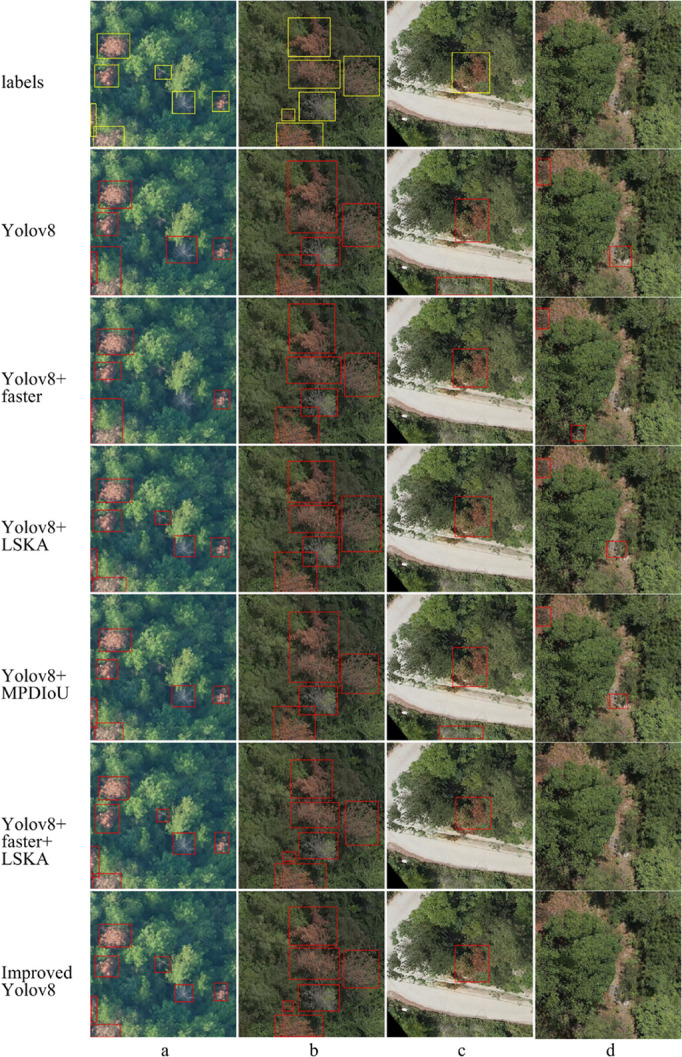
Ablation study visualization results.

**Fig 11:**
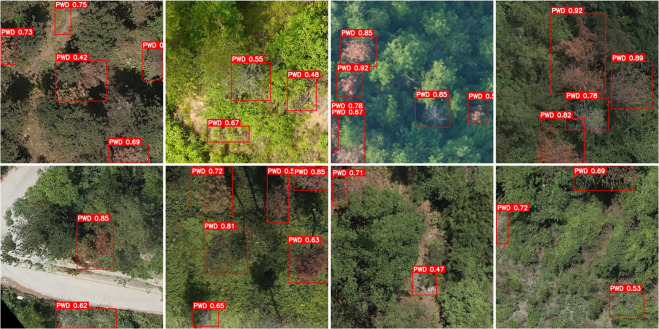
YOLOv8 identification results.

**Fig 12:**
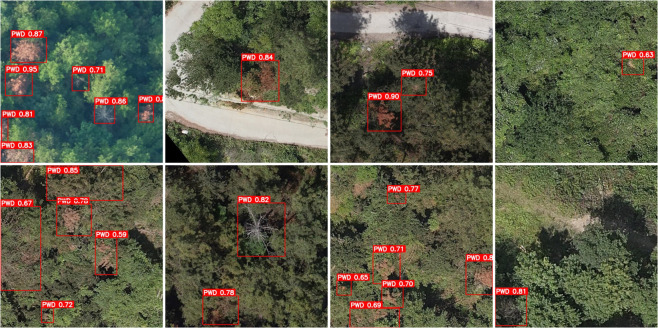
FLMP-YOLOv8 identification results.

The visualization results of the ablation experiment are shown in Fig 10; The identification results of the baseline YOLOv8 model are shown in Fig 11, while the identification results of the proposed FLMP-YOLOv8 model are shown in Fig 12.

As shown in Fig 10, the visualization results of ablation experiments under different lighting conditions demonstrate the performance of each model variant. The original YOLOv8 model exhibits significant omissions in detecting small targets and is prone to false positives in bare land areas. The integration of the FasterBlock module improves detection speed but contributes limited enhancement to accuracy. With the addition of the LSKA module, the model shows a notable improvement in recognizing small targets and achieves more stable detection under background interference. Furthermore, the MPDIoU loss function enhances the localization accuracy of bounding boxes. As a result, the proposed FLMP-YOLOv8 model demonstrates stronger target discrimination and environmental adaptability, enabling more accurate detection of disease-infected trees of various sizes while effectively suppressing background interference, thereby significantly improving overall detection performance. Figs 11 and 12 present the comparative detection results of YOLOv8 and FLMP-YOLOv8 in the task of pine wild Disease -infected trees identification. It can be observed that FLMP-YOLOv8 exhibits more stable performance under complex background conditions, showing greater robustness in regions such as bare land and interforest gaps, with significantly reduced false detections. Additionally, the model demonstrates enhanced capability in detecting small-sized targets, successfully identifying instances that were missed by the YOLOv8 model. The predicted bounding boxes from FLMP-YOLOv8 also show consistently higher confidence scores across multiple samples, indicating improved reliability and accuracy in the detection task. These results validate the effectiveness of the proposed architectural improvements and loss function design.

## Discussion

In addition to earlier works discussed above, numerous recent studies have explored enhancements in deep-learning-based methods for pine wilt disease (PWD) detection, offering important points of comparison to assess the strengths and trade-offs of FLMP-YOLOv8.

Traditional machine learning approaches remain useful in certain contexts. For instance, in central Portugal, Iordache *et al*. (2020) used Random Forest classifiers on ultra-high-resolution multispectral and hyperspectral data and achieved over 0.91 overall accuracy [9]. Yu *et al*. (2021) fused hyperspectral imaging and UAV-based LiDAR data to improve stage-level identification of infection at the individual tree level [10]. Oide *et al*. (2022) compared several classifiers (Logistic Regression, SVM, KNN, RF, ANN) over both RGB and HSV color spaces in Japan, finding ANN with HSV inputs provided top performance among traditional methods [12]. These approaches generally offer strong interpretability and perform well with modest sample sizes, but they often depend on expensive sensors, heavier preprocessing, and carefully engineered features, which may limit robustness across different forest terrains and lighting conditions.

More recently, a number of works have improved one- and two-stage object detectors with attention modules, transformer components, or customized loss functions. Examples include Mask R-CNN variants enhanced with modules such as ConvNeXt, PA-FPN, and normalization techniques (GN, WS) to improve instance segmentation and detection under high resolution images [13]; multichannel CNNs using multispectral bands (e.g., RGB + NIR + Red-Edge) plus vegetation indices (such as NDRE) to enhance detection in complex backgrounds [14]; “lightweight” models designed for speed and computational efficiency (e.g., replacing backbones with MobileNetV2, using attention modules like CBAM or CA) [18].

When compared with these studies, FLMP-YOLOv8 shows several advantages:

Sensor and data modality: Unlike many approaches relying on multispectral or hyperspectral sensors, FLMP-YOLOv8 performs well using standard RGB UAV imagery. This reduces equipment cost and simplifies data acquisition and preprocessing.

Feature aggregation and architectural improvements: The inclusion of FasterBlock and LSKAttention enhances multi-scale feature extraction and suppresses background interference (e.g. similar textures, shadows, variable lighting) more effectively than many lightweight attention-augmented models that primarily focus on channel attention or spectral bands.

Localization and loss design: The use of MPDIoU loss contributes to more accurate bounding box regression, especially for small or obscure disease regions. Compared to models using standard IoU or other loss functions, this results in better overlap with ground truth in challenging conditions.

Speed-accuracy trade-off: Some “fast” models sacrifice localization precision or miss small targets, while many high-precision two-stage or segmentation models are too slow for large-scale UAV monitoring. FLMP-YOLOv8 manages to maintain high detection speed (over 80 FPS) while still obtaining strong mAP@0.5 ( 87%), showing an excellent balance suited for real-world forest surveillance.

Nevertheless, these comparisons are not without caveats. Differences in datasets (e.g., resolution, disease stage, forest type), degree of annotation (tree-level vs canopy vs pixel/segmentation), and environmental conditions mean that direct numerical comparisons must be interpreted cautiously. Future work could include benchmarking FLMP-YOLOv8 across multiple publicly available datasets, integrating some spectral or temporal information (video or multi-date imagery), and exploring lightweight attention or transformer modules that further improve sensitivity to early-stage symptoms.

## Conclusion

This study aims to enhance the detection accuracy and speed of pine wood nematode-infected trees in complex forest environments by improving the YOLOv8 algorithm. Specifically, the Bottleneck in the C2f module is replaced with the FarsterBlock structure to optimize feature extraction and enhance detection speed. The addition of LSKAttention to the Spatial Pyramid Pooling Layer (SPPF) effectively improves the ability of the SPPF module to aggregate features at multiple scales. Furthermore, the introduction of the MPDIoU bounding box regression loss function brings the predicted boxes closer to the ground truth, further Improving the detection accuracy of the model. A series of experimental results show that this method outperforms the original framework and other algorithms, achieving a dual improvement in both detection accuracy and speed for identifying pine wood nematode-infected trees. Compared to the baseline YOLOv8 model, the improved algorithm achieves a 2.2% increase in precision (P), a 0.6% increase in recall (R), and a 2.0% increase in mAP@0.5 (mean average precision), with detection speed significantly improving from 16.31 to 81.79. The increase in detection speed significantly reduces the model’s reliance on hardware resources, enhancing the economic efficiency and convenience of detecting pine wood nematode-infected trees. Meanwhile, the high-precision detection structure is crucial for identifying and isolating infected trees, helping to reduce the spread of pine wood nematode disease and protect forest ecosystems.

However, despite the positive results, further research is warranted. Given the varying manifestations of pinewood nematode disease across regions, studying the model’s generalization ability across diverse environments is crucial. Future research will focus on validating and enhancing the model’s generalization ability to improve detection performance. Moreover, integrating mobile platforms like drones to develop portable and automated detection systems will facilitate broader real-world applications.

In conclusion, this study successfully enhances the accuracy and speed of detecting pinewood nematode-infected trees by improving the YOLOv8 model. This achievement has significant implications for detecting and controlling pinewood nematode disease and provides new ideas and methods for intelligent monitoring of forest pests and diseases.
